# OP64 Exploring The Environment And Capacity Of South African Citizen Actors To Contribute To Health Technology Assessment Processes, Policy Development, And Institutionalization

**DOI:** 10.1017/S0266462325101219

**Published:** 2025-12-29

**Authors:** Debjani Mueller, Lauren Pretorius, Anke-Peggy Holtorf, Henrietta Van Kramberg

## Abstract

**Introduction:**

A legislation, regulation, and policy review of limited South African legislative framework of health technology assessment (HTA) was undertaken to identify opportunities to advocate for required legislation reform or development to entrench principles and processes for patient and citizen involvement (PCI). We reviewed and mapped the capacity, knowledge, and skills of South African patient and citizen advocacy actors to actively advocate in developing regulations and policy in the National Health Insurance landscape.

**Methods:**

We used a literature scan and analysis of relevant national and international documentation, including reports of primary and secondary data analysis to aid in the identification of current gaps in, and opportunities for, PCI in HTA in South Africa. Data sources included resolutions, legislation, regulations, government policy, and technical reports gleaned from websites of relevant agencies and experts. An electronic survey was distributed to 213 patient and citizen actors. The survey aimed to establish the capacity, knowledge, and skills of South African patient and citizen advocacy actors and organizations across multiple disease areas, predominately non-communicable diseases.

**Results:**

Legislation and policy documents indicated engagement initiatives are located at “involvement” or “consultation” stages of the engagement continuum. Patient advocacy groups participated in raising awareness of disease burden, advocating for adoption of specific technology, or utilizing legal channels to compel coverage arrangements for specific therapies.

The barriers and challenges observed were:

- lack of skills, training, and education;
- insufficient information regarding roles and responsibilities;
- lack of logistical or financial support; and
- lack of integrated strategy.

Possible dimensions for capacity building include select starting points that build on research partners’ strengths, meeting the immediate needs of governments, and contributing to longer-term goals.

**Conclusions:**

Results indicated non-involvement of key actors in crucial healthcare processes. Actors were adamant that they would have greater influence at the provincial or national level to make meaningful contributions. Existing legislative and policy frameworks do not include PCI capacity building strategies. We can only conclude that once healthcare processes are inclusive, PCI will become mandatory.

